# Delayed Neuroinflammation Mimicking Stroke After Flow-Diverting Stent Placement: A Case Report on Diagnostic and Therapeutic Challenges

**DOI:** 10.7759/cureus.92443

**Published:** 2025-09-16

**Authors:** Muhammad Arslan Ghous, Faisal Bashir Chaudhry, Ahmed Malik Abuelgasim Malik, George Horton

**Affiliations:** 1 Stroke Medicine, University Hospitals of North Midlands, Stoke-on-Trent, GBR; 2 Internal Medicine, University Hospitals of North Midlands, Stoke-on-Trent, GBR

**Keywords:** corticosteroid treatment, diagnostic challenge, flow-diverting stent, intracranial aneurysm, multidisciplinary evaluation, neuroinflammation, non-ischaemic cerebral enhancing (nice) lesions, stroke mimic

## Abstract

Flow-diverting stents are increasingly used in the management of complex intracranial aneurysms, offering superior occlusion rates compared to conventional techniques; however, rare complications such as neuroinflammation can mimic more common conditions like stroke, creating significant diagnostic challenges.

We report a case of a 50-year-old woman with multiple intracranial aneurysms, hypertension, hypercholesterolaemia, and a prior stroke who underwent flow-diverting stent placement for a recurrent right posterior communicating artery aneurysm. Four weeks post procedure, she presented with severe headache, photophobia, neck stiffness, and unilateral weakness. Initial imaging suggested acute ischaemic stroke or metastatic disease, but serial MRI demonstrated a dynamic pattern of progressive and regressive lesions. Multidisciplinary evaluation ultimately identified neuroinflammation, specifically non-ischaemic cerebral enhancing (NICE) lesions, a rare stent-related complication. Treatment with corticosteroids led to marked clinical and radiological resolution.

This case underscores the typical features of post-stent neuroinflammation, such as delayed onset, stroke-like symptoms, and steroid responsiveness, while highlighting its diagnostic complexity due to overlapping presentations with ischaemic events. It also suggests that prior interventions may confer increased risk, emphasising the importance of serial imaging and multidisciplinary collaboration in achieving accurate diagnosis and optimal patient outcomes.

## Introduction

Flow-diverting stents have revolutionised the endovascular treatment of intracranial aneurysms, particularly those that are large, wide-necked, fusiform, or located in anatomically challenging segments where conventional microsurgical clipping or endovascular coiling may be technically unfeasible or associated with high recurrence rates [1,2]. These devices are designed to redirect laminar blood flow along the parent vessel, thereby reducing intra-aneurysmal circulation, promoting gradual thrombosis, and facilitating endothelialisation across the aneurysm neck while preserving patency of the parent artery. Over the past decade, flow diversion has demonstrated superior long-term occlusion rates, reduced need for retreatment, and favourable safety profiles compared to traditional approaches [1,3].

Despite these advantages, their use is not without risks. Well-recognised complications include periprocedural ischaemic stroke, in-stent thrombosis, delayed aneurysm rupture, and haemorrhagic events. In contrast, delayed neuroinflammatory reactions following stent placement, particularly those manifesting as non-ischaemic cerebral enhancing (NICE) lesions, remain exceedingly rare, with an estimated incidence of approximately 1% [4,5]. The underlying pathophysiology is incompletely understood, though hypotheses include a delayed immune-mediated response to stent components, alteration of local haemodynamics, and perivascular inflammation triggered by endothelial disruption.

Such inflammatory presentations can mimic common post-stent complications, most notably ischaemic stroke, due to overlapping neurological features such as focal deficits, headache, and seizure activity [6,7]. This resemblance often results in diagnostic uncertainty, necessitating careful interpretation of neuroimaging and integration of serial MRI findings with clinical evolution [6,7]. In many reported cases, a delayed onset, multifocal enhancing lesions, and responsiveness to corticosteroid therapy help distinguish neuroinflammation from ischaemic or neoplastic processes [5-7]. Here, we describe a case of a 50-year-old woman with a history of multiple intracranial aneurysms who underwent flow-diverting stent placement for a recurrent right posterior communicating artery aneurysm and subsequently developed delayed-onset, stroke-like symptoms. The diagnosis of stent-related neuroinflammation was established through multidisciplinary evaluation, and timely corticosteroid therapy resulted in marked clinical and radiological improvement. This case not only adds to the limited literature on this unusual complication but also highlights the importance of early recognition, appropriate imaging follow-up, and multidisciplinary collaboration in optimising patient outcomes.

## Case presentation

A 50-year-old woman with a history of hypertension, hypercholesterolemia, pre-diabetes, migraine, prior lacunar ischaemic stroke, small vessel disease, and functional neurological disorder (FND) with left-sided symptoms presented for evaluation. She had a 35-year smoking history (15 cigarettes daily, transitioned to vaping) and an unconfirmed nickel allergy.

In October 2022, she experienced transient right-sided weakness and dysarthria. Imaging excluded acute stroke but incidentally identified three intracranial aneurysms: an 8 mm basilar tip aneurysm, a 6 mm right posterior communicating artery (PCOM) aneurysm, and a right cavernous internal carotid artery (ICA) aneurysm. The neurovascular multidisciplinary team (MDT) prioritised embolisation of the basilar tip aneurysm using a contour device in June 2023, followed by treatment of the right PCOM aneurysm with four Hydrocoils in July 2023, achieving occlusion with a small neck remnant.

Follow-up imaging in March 2024 revealed recurrence of the right PCOM aneurysm, which had enlarged by November 2024, necessitating urgent intervention. On April 23, 2025, the aneurysm underwent coil embolisation with deployment of a 4.25 mm × 14 mm Pipeline flow-diverter stent across the right paraclinoid ICA, achieving stasis within the aneurysm and preserving cerebral blood flow without complications.

**Figure 1:**
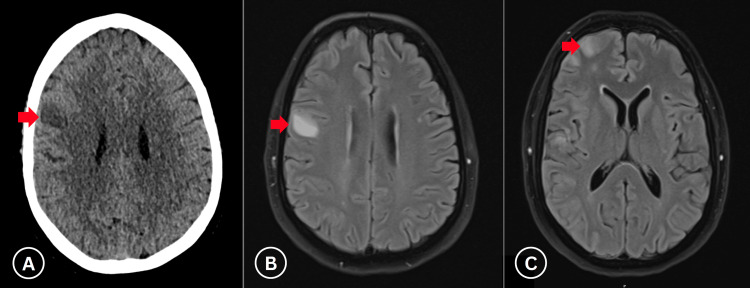
Initial imaging following symptom onset. (A) Non-contrast CT shows right frontal and peri-rolandic hypodensity consistent with acute middle cerebral artery territory infarction. (B, C) MRI demonstrates multiple enhancing cortical lesions in the right hemisphere (red arrows), initially suspicious for metastatic disease.

Three weeks later, in mid-May 2025, the patient presented multiple times with severe headache (10/10), photophobia, neck stiffness, and left-sided hemiparesis and hypoesthesia affecting both extremities. Initial non-contrast computed tomography (CT) of the head showed right frontal and peri-rolandic cortical hypodensity, suggestive of an acute infarct in the right middle cerebral artery (MCA) territory, absent on prior magnetic resonance imaging (MRI). Concurrent CT angiography confirmed stent patency without additional abnormalities. Subsequent MRI with magnetic resonance angiography (MRA) and diffusion-weighted imaging revealed multiple right hemispheric cortical and pial-based lesions, initially raising suspicion of metastatic disease (Figure 1). Differential diagnoses included acute ischaemic stroke and metastatic disease.

Due to complexity and diagnostic dilemma, this case was discussed in multiple multidisciplinary meetings. Neuro-oncology MDT concluded that lesions did not fit with metastatic disease but suggested multiple embolic strokes as a possible underlying aetiology. The stroke neuroradiology meeting favoured neuroinflammation, potentially due to a foreign body reaction to the stent coating. Given the patient's unconfirmed nickel allergy and the stent's nickel content, an allergic reaction was hypothesised; however, following specialised immunology input, it was deemed unlikely.

**Figure 2:**
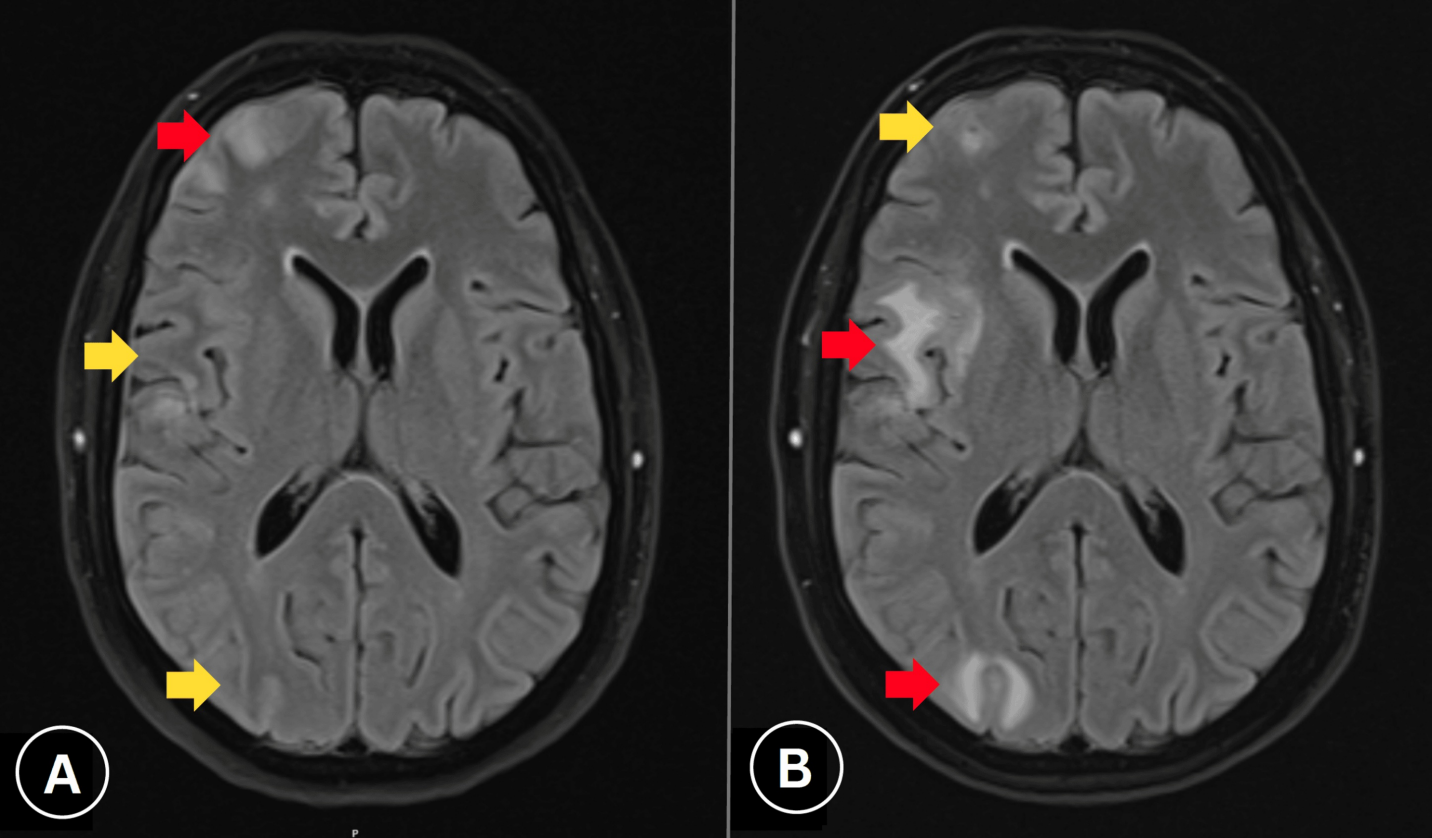
Serial MRI demonstrating lesion evolution. (A) Baseline MRI showing right hemispheric cortical lesion distribution. (B) Two-week follow-up demonstrates progression in parietal-occipital regions (red arrows) with concurrent resolution of frontal enhancement (yellow arrows), consistent with inflammatory rather than neoplastic aetiology.

**Figure 3:**
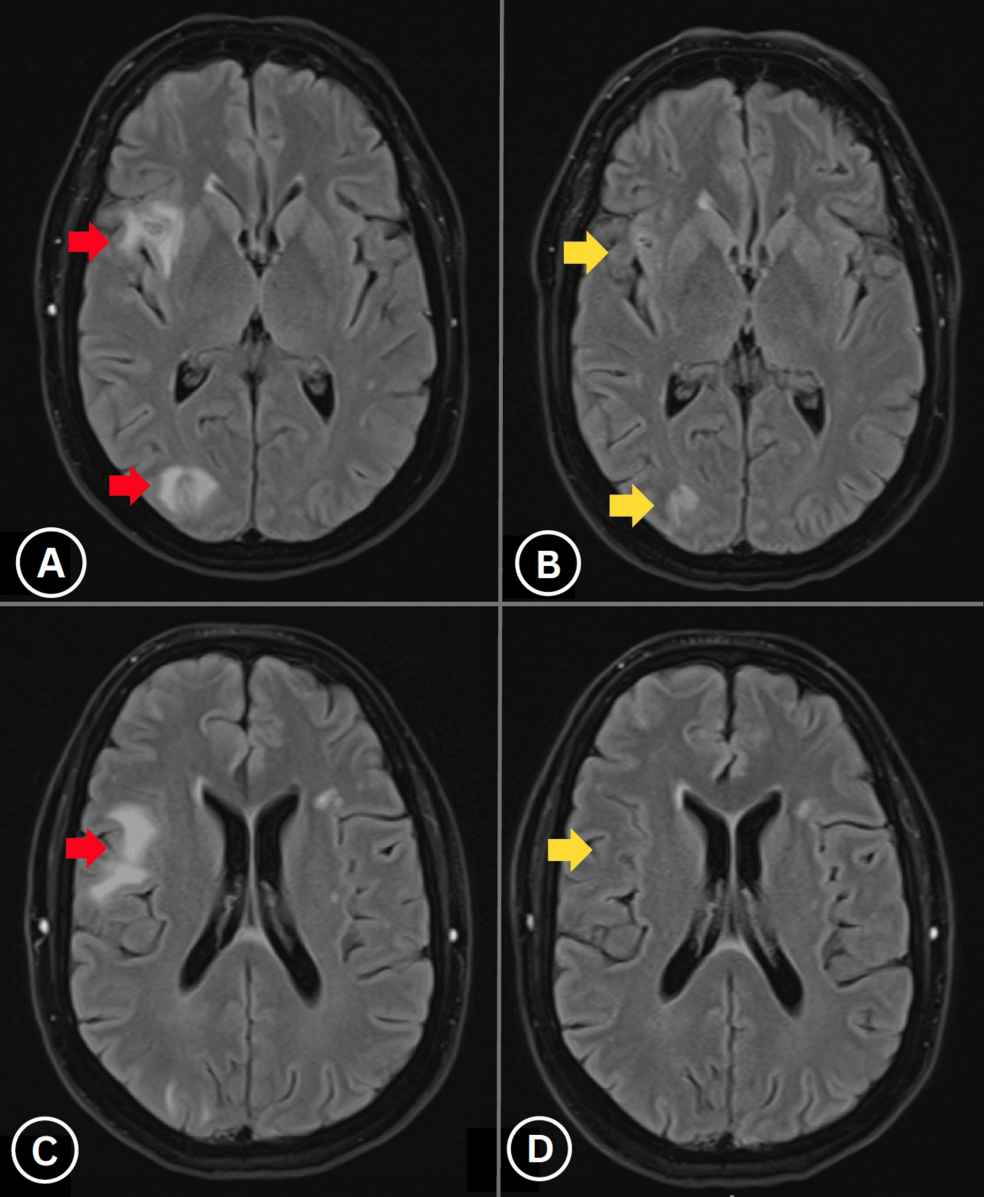
Post-corticosteroid treatment response. (A, C) Pre-treatment MRI at two weeks showing inflammatory lesion progression. (B, D) Post-corticosteroid MRI at five weeks demonstrates significant lesional regression (red arrows), confirming steroid-responsive inflammatory process.

Due to diagnostic uncertainty, serial imaging was performed. A repeat MRI two weeks later showed progression of right parietal and occipital lesions with regression of lesions in the right inferior frontal gyrus and motor hand knob area (Figure 2), supporting an inflammatory aetiology. Immunosuppressive therapy with prednisolone (50 mg daily for one week) was initiated. A follow-up MRI three weeks later demonstrated a marked reduction in right hemispheric lesional enhancement, particularly in parietal and occipital regions, with no new lesions (Figure 3).

We concluded that these findings aligned with non-ischaemic cerebral enhancement, an inflammatory condition [5,6]. Corticosteroids were tapered, and the patient resumed normal activities on dual antiplatelet therapy (DAPT). This case highlights neuroinflammation as a rare, delayed complication of flow-diverting stents, emphasising the need for heightened clinical suspicion in similar presentations.

## Discussion

Flow-diverting stents have transformed the management of complex intracranial aneurysms by redirecting blood flow to promote aneurysm occlusion while preserving parent vessel integrity [1,3]. Despite their efficacy, complications such as periprocedural stroke, in-stent thrombosis, and, more rarely, neuroinflammation can occur.

This case report describes a 50-year-old female who developed neuroinflammation four weeks after the placement of a flow-diverting stent for a recurrent right PCOM aneurysm, initially mimicking an acute ischaemic stroke. Through serial imaging and MDT discussions, the diagnosis of stent-related neuroinflammation was established, highlighting a rare but clinically significant complication that remains underreported in the literature.

Neuroinflammation following flow-diverting stent placement, particularly in the form of NICE lesions, has an estimated incidence of 1% in recent multi-centre studies [4,5]. These lesions typically present weeks to months post procedure and can be misdiagnosed due to their overlapping clinical and radiological features with stroke or metastatic disease. In our case, the patient presented with severe headache, photophobia, neck stiffness, and transient left-sided weakness four weeks after stent placement, consistent with the timing and symptom spectrum of NICE lesions, which range from asymptomatic to severe neurological deficits. Radiologically, serial MRI revealed multiple right hemispheric cortical and pial-based lesions with a mixed pattern of progression and regression, a hallmark of inflammatory processes rather than ischaemia. This aligns with the literature, where NICE lesions are characterised by enhancing lesions without corresponding diffusion restriction, often resolving with corticosteroid therapy [6,7].

Our case fits within the broader context of stent-related neuroinflammation, particularly NICE lesions, as described in recent studies. The use of a flow-diverting stent in our patient is notable, given its association with a higher incidence of NICE lesions (3.7%) compared to other devices [4]. The patient’s history of multiple prior endovascular treatments [8] and cardiovascular risk factors may have contributed to an increased inflammatory predisposition, suggesting that individual characteristics could influence the development of this complication. The patient’s presentation at four weeks post procedure falls within the reported onset range for NICE lesions (two weeks to 12 months), and her favourable response to corticosteroids mirrors the outcomes observed in approximately 40% of NICE cases treated with steroids [5].

The diagnostic process in this case underscores the challenges of distinguishing neuroinflammation from more common post-procedural events. Initial computed CT and MRI findings were suggestive of stroke or metastasis, respectively, and it was only through repeated imaging and MDT collaboration that the dynamic nature of the lesions, indicative of inflammation, was recognised. This mirrors the diagnostic difficulties reported in the literature, where NICE lesions are often misdiagnosed due to their rarity and atypical presentation [6,7]. Our case reinforces the importance of serial imaging and a high index of suspicion for neuroinflammation in patients with new neurological symptoms weeks to months after flow-diverting stent placement, particularly when imaging is inconsistent with typical ischaemic patterns.

Management of stent-related neuroinflammation typically involves corticosteroid therapy, as demonstrated in our case. The patient was treated with prednisolone 50 mg daily, tapered over time, leading to significant clinical and radiological improvement. This outcome is consistent with the literature, where steroids have been effective in resolving or improving NICE lesions in the majority of cases [5]. Importantly, DAPT was maintained, consistent with the current understanding that neuroinflammation does not typically necessitate changes in antithrombotic regimens. This case thus supports the current treatment paradigm for NICE lesions while providing a detailed example of steroid dosing and tapering in clinical practice [2,9,10].

## Conclusions

This case report contributes to the limited but growing body of literature on neuroinflammatory complications following flow-diverting stent placement. By detailing the clinical course, diagnostic challenges, and successful management of a rare case of stent-related neuroinflammation, our report emphasises the need for heightened awareness of this complication among clinicians. The use of serial imaging and MDT discussions was pivotal in reaching the correct diagnosis, offering a practical framework for similar cases. As flow-diverting stents remain a cornerstone in treating complex aneurysms, recognising and managing rare complications like neuroinflammation is essential to optimising patient outcomes. This case adds valuable insights to the literature and underscores the importance of ongoing investigation into device-specific risks and long-term follow-up strategies.

Future studies should explore whether patient-specific risk factors, such as previous endovascular procedures, cardiovascular comorbidities, or metal sensitivities, can help predict neuroinflammatory responses to stent placement. Additionally, identifying characteristic imaging features in the early stages could improve diagnostic accuracy and reduce unnecessary treatment delays.
